# Modeling the evolution of SARS-CoV-2 under non-pharmaceutical interventions and testing

**DOI:** 10.1093/emph/eoac013

**Published:** 2022-04-18

**Authors:** Yael Gurevich, Yoav Ram, Lilach Hadany

**Affiliations:** 1 Faculty of Life Sciences, School of Plant Sciences and Food Security, Tel-Aviv University, Tel-Aviv 6997801, Israel; 2 Faculty of Life Sciences, School of Zoology, Tel-Aviv University, Tel-Aviv 6997801, Israel

**Keywords:** evolution, non-pharmaceutical interventions, SARS-CoV-2, coronavirus

## Abstract

**Background and objectives:**

Social and behavioral non-pharmaceutical interventions (NPIs), such as mask-wearing, social distancing and travel restrictions, as well as diagnostic tests, have been broadly implemented in response to the COVID-19 pandemic. Epidemiological models and data analysis affirm that wide adoption of NPIs helps to control the pandemic. However, SARS-CoV-2 has extensively demonstrated its ability to evolve. Therefore, it is crucial to examine how NPIs may affect the evolution of the virus. Such evolution could have important effects on the spread and impact of the pandemic.

**Methodology:**

We used evo-epidemiological models to examine the effect of NPIs and testing on two evolutionary trajectories for SARS-CoV-2: attenuation and test evasion.

**Results:**

Our results show that when stronger measures are taken, selection may act to reduce disease severity. Additionally, the timely application of NPIs could significantly affect the competition between viral strains, favoring the milder strain. Furthermore, a higher testing rate can select for a test-evasive viral strain, even if that strain is less infectious than the detectable competing strain. Importantly, if a less detectable strain evolves, epidemiological metrics such as confirmed daily cases may distort our assessment of the pandemic.

**Conclusions and implications:**

Our results highlight the important implications NPIs can have on the evolution of SARS-CoV-2.

**Lay Summary:**

We used evo-epidemiological models to examine the effect of non-pharmaceutical interventions and testing on two evolutionary trajectories for SARS-CoV-2: attenuation and test evasion. Our results show that when stronger measures are taken, selection may act to reduce disease severity.

## INTRODUCTION

Social and behavioral non-pharmaceutical interventions (NPIs) have been broadly applied to contain the COVID-19 pandemic. These interventions include use of face masks, implementation of social distancing, closure of educational institutions, individual movement restrictions and quarantining cases confirmed using RT-PCR or serological testing. The role of NPIs in controlling the COVID-19 pandemic has been studied extensively [1]. Epidemiological models have been used to assess the impact of these NPIs on the pandemic, aiming to forecast the levels of infection [2], hospitalization [3] and mortality [4]. Both theoretical models and data analysis affirm that wide and early adoption of interventions, such as limiting social contacts and wearing face masks, helps to control the pandemic [1]. However, SARS-CoV-2 has broadly demonstrated its ability to evolve [5]: it has been suggested that a mutation conferring ability to infect humans [5] preceded its transmission to humans from bats [5]. Similar to other RNA viruses [6], the mutation rate of SARS-CoV-2 is estimated at ∼10^−6^ per site/cycle, relatively high [7] (although lower than Influenza [8]). Additionally, there is already significant variation in the viral population [9] due to a high rate of recombination [5], a very high number of copies produced in each infection [7], a rapid replication cycle (around 10 h [7]) and the large effective size of the SARS-CoV-2 population. This variation can potentially lead to adaptive evolution [9], as seen before, e.g. in Influenza [10], HIV [11] and Ebola [12].

Because the virus only recently emerged in humans, further adaptation of SARS-CoV-2 to its new host is likely. Indeed, new strains have recently emerged carrying mutations that may increase transmission, lower detectability, and perhaps even reduce vaccine efficiency [9]. Importantly, by limiting the transmission of the virus, NPIs may exert strong selection on SARS-CoV-2 [13]. Hence, it is crucial to examine how NPIs could affect the evolution of the virus. Such evolution may have important effects on the spread and control of the pandemic. To examine how the virus may evolve in response to NPIs, we have developed evo-epidemiological models that track both the infection status of the human hosts and the strain of the infecting virus. We use these models to examine how NPIs are expected to affect two evolutionary trajectories for SARS-CoV-2: attenuation and test evasion.

### Attenuation

An important epidemiological feature of the virus is the high frequency of asymptomatic infections [14]. It is suggested that asymptomatic individuals are infectious [15] and can transmit the disease but are less infectious than those who are symptomatic [15]. Due to limited resources, the COVID-19 testing policy in many countries does not include routine screening of asymptomatic individuals, unless they have been in direct contact with a confirmed case or are routinely exposed to infected individuals (e.g. health workers). Thus, these asymptomatic cases largely go undetected. Asymptomatic infection allows the individual to maintain their normal routine and social contact levels throughout the entire course of the infection, thus potentially producing many secondary infections. The tendency to develop asymptomatic infection is affected both by the individual characteristics, such as prior health status [16], and by the virus itself. As asymptomatic cases are less likely to be diagnosed and isolated, we hypothesized that a decrease in the frequency of symptomatic cases can be favored by selection, leading to the evolution of an attenuated pathogen [17]. For example, a mutation causing decreased viral load may cause a higher frequency of asymptomatic cases and milder disease. However, asymptomatic individuals are likely less infectious [15], and if the relative transmissibility of asymptomatic cases is low enough, the more virulent strain may evolve. We use the term ‘virulence’ to describe the severity of disease produced by the virus [18, 19]. Increased awareness to the epidemic and application of NPIs may select for further increase in the frequency of asymptomatic infections. NPIs change the overall infection rate by reducing the number of contacts per individual, hence we expect that NPIs will have an important role in determining the outcome of competitions between attenuated and virulent strains.

### Test evasion

An active COVID-19 infection can be diagnosed using an RT-PCR test [20] on a nasopharyngeal swab specimen, detecting specific sites in the viral genome, or a rapid antigen test [21]. The detected sites were chosen such that they are critical for virus function [22]. COVID-19 tests have been evaluated for their sensitivity, the expected fraction of infected individuals who receive a positive test result, and specificity, the fraction of uninfected individuals who erroneously receive a positive test result [20]. The conditions under which individuals are tested may differ between and even within different countries [23]. Given that individuals who receive a positive test result are isolated until recovery, largescale testing can exert strong selection pressure on the virus. While a false negative result on a COVID-19 test can also be caused by human error in test administration [24], we assume that the human factors are similar for both strains. The detectability of the virus may be directly under selection, potentially favoring two classes of mutants: (i) mutants presenting atypical infections [25], including affecting different age groups (e.g. children) or different tissues [25] (e.g. gastrointestinal system, heart and liver infections). Undiagnosed COVID-19 patients may not be quarantined even when sick—heart disease, e.g. is not usually infectious—and therefore might infect others, including healthcare workers and other patients. (ii) As tests are used to determine who must be quarantined, studies have shown that mutations in the viral genome could affect the accuracy of RT-PCR-based detection assays [22] and antigen tests [26]. Viruses with modifications in the RNA sequence used for the test could be favored by natural selection. Although tests can be modified to detect different viral strains, we assume a delay between the evolution of the virus and a wide availability of adapted tests. We hypothesize that when testing is frequent and NPIs are significant, selection could favor strains that are harder to detect, even at the cost of lower transmissibility.

## MODEL

**Figure 1.**
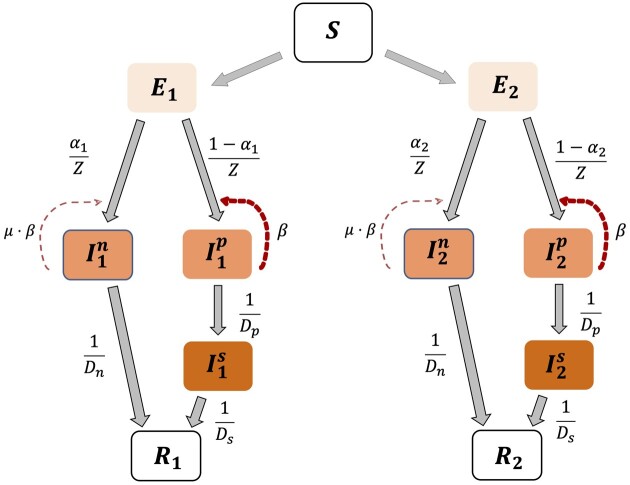
Evo-epidemiological model. Our model follows two viral strains simultaneously spreading in an initially susceptible population. Susceptible (S) individuals become exposed (E) after contact with an infected individual with rate β, μ⋅β for pre-isolated and non-isolated individuals, respectively. Exposed individuals (E) undergo an incubation period during which they are not infectious. After an average incubation period of Z days, exposed individuals become infected, and are either non-isolated ($I^{n}$) or pre-isolated ($I^{p}$) or, with probability α and (1- α), respectively. Non-isolated individuals ($I^{a}$) are infectious but not isolated from disease onset until recovery, e.g. because they are asymptomatic. Pre-isolated individuals ($I^{p}$) are infectious and may be asymptomatic or exhibit mild symptoms for several days, after which they exhibit clinical manifestation of the disease ($I^{s}$) and are therefore isolated. Isolated and non-isolated cases become recovered (R) after an average of $D_{s}$ and $D_{a}$ days, respectively.

We use an SEIR compartmental epidemic model. The model follows two viral strains simultaneously spreading in an initially susceptible population (Fig. 1). We define non-isolated individuals ($I^{a}$) to be those who are infectious but not isolated from disease onset until recovery, e.g. because they are asymptomatic. We define pre-isolated individuals ($I^{p}$) to be those who are infectious and may be asymptomatic or exhibit mild symptoms for several days, after which they exhibit clinical manifestation of the disease ($I^{s}$) and are therefore isolated. We neglect births and deaths due to non-disease related causes and assume no superinfection and total cross-immunity, such that recovered individuals from either strain are immune to both strains. For COVID-19, the latter is likely true in some strains for at least several months [27]. Thus, we divide the host population to susceptible individuals (S), individuals exposed to one of the strains ($E_{1},E_{2}$ for Strain 1 and Strain 2, respectively), infected individuals, including non-isolated ($I_{1}^{n}$, $I_{2}^{n}$), pre-isolated ($I_{1}^{p},I_{2}^{p}$), and isolated individuals ($I_{1}^{s},I_{2}^{s}$) and ‘removed’ individuals ($R_{1},R_{2}$), which effectively include both recovered individuals and fatalities. The initial conditions for the two viral strains are identical, and we consider a small number of exposed and infected individuals. Strain 1 is the ‘virulent’ strain, and Strain 2 is ‘attenuated’, resulting in weaker symptoms and a higher fraction ($\alpha_{i}$) of non-isolated cases ($\alpha_{2}>\alpha_{1}$).

Susceptible individuals become exposed through contact with an infected individual. Let β be the transmission rate for pre-isolated individuals. The distinction between the transmission rates of the different classes of infected individuals is central: non-isolated individuals are assumed to be less infectious than those who are pre-isolated, e.g. due to lower viral load, and the transmission rate can be very low for isolated cases, due to a low contact rate. The parameter μ defines relative transmission rate of non-isolated infected $\left(\mu \cdot \beta \right)$ individuals in comparison to pre-isolated individuals $\left(\beta \right)$.

The basic model is described by the following equations (i=1,2):

(1) $$\frac{dS}{dt}=-\frac{S}{N}\cdot \beta \cdot (I_{1}^{p}+\mu \cdot I_{1}^{n}+I_{2}^{p}+\mu \cdot I_{2}^{n})$$

(2) $$\frac{dE_{i}}{dt}=\frac{S}{N}\cdot \beta \cdot \left(I_{i}^{p}+\mu \cdot I_{i}^{n}\right)-\frac{E_{i}}{Z}$$

(3) $$\frac{dI_{i}^{p}}{dt}={(1-\alpha }_{i})\cdot \frac{E_{i}}{Z}-\frac{I_{i}^{p}}{D_{p}}$$

(4) $$\frac{dI_{i}^{s}}{dt}=\frac{I_{i}^{p}}{D_{p}}-\frac{I_{i}^{s}}{D_{s}}$$

(5) $$\frac{dI_{i}^{n}}{dt}=\alpha_{i}\cdot \frac{E_{i}}{Z}-\frac{I_{i}^{n}}{D_{n}}$$

(6) $$\frac{dR_{i}}{dt}=\frac{I_{i}^{n}}{D_{n}}+\frac{I_{i}^{s}}{D_{s}}$$

Note that $S,E_{1},E_{2},\;I_{1}^{p},I_{2}^{p},I_{1}^{s},I_{2}^{s},I_{1}^{n},I_{2}^{n},R_{1},R_{2}\geq 0$ and $S+E_{1}+E_{2}+\;I_{1}^{p}+I_{2}^{p}+I_{1}^{s}+I_{2}^{s}+I_{1}^{n}$ + $I_{2}^{n}+R_{1}+R_{2}=N$, where *N* is the constant host population size.

### Basic reproduction number and stability analysis

The basic reproduction number $R_{0}$ of an epidemic can be interpreted as the expected number of secondary cases produced by a typical infected individual in a completely susceptible population [28]. It is associated with the transmissibility of the epidemic in a new host population. We define $R_{0}^{i},\;(i=1,2)$ as the basic reproductive number for each of the viral strains in our system.

We applied the next-generation approach [29] to compute the basic reproduction number. The infected compartments are $E_{1},E_{2},\;I_{1}^{p},I_{2}^{p},I_{1}^{s},I_{2}^{s},I_{1}^{n},I_{2}^{n}$. The next-generation (i.e. transition) matrix is defined as $FV^{-1}$, where *F* describes the production of new infected and *V* describes transitions between infected states. The matrix has two non-zero eigenvalues, corresponding to the reproductive numbers for each strain: $R_{0}^{i}=\underset{\mathrm{pre}-\mathrm{isolated}}{\underbrace{\left(1-\alpha_{i}\right){\cdot D}_{p}\cdot \beta }}+\underset{\mathrm{non}-\mathrm{isolated}}{\underbrace{{\alpha_{i}\cdot D}_{n}\cdot \mu \cdot \beta }}$ (see details in Supplementary data). Only the pre-isolated and the non-isolated compartments contribute to $R_{0}$, as individuals in the other compartments do not produce new infections. This can be interpreted as the probability that a given individual is either pre-isolated $\left(\alpha_{i}\right)$ or non-isolated $\left(1-\alpha_{i}\right)$, multiplied by the number of days from beginning of infectiousness until recovery $(D_{p},\;D_{a}$ for pre-isolated and non-isolated, respectively) and the transmission rate (β, μ⋅β for pre-isolated and non-isolated, respectively). In the case of the test-evasive strain, we assume that a proportion p of the population is tested for SARS-CoV-2 infection every day and that the test-evasive strain incurs a cost of infectiousness, c>0, such that its transmission rate is reduced by a factor of (1-c). The reproduction number for each strain is $R_{0}^{1}=\underset{\mathrm{pre}-\mathrm{isolated}}{\underbrace{\frac{\left(1-\alpha_{i}\right)\cdot \beta }{\left(p\cdot d_{i}\;+\frac{1}{D_{p}}\right)}}}+\underset{\mathrm{non}-\mathrm{isolated}}{\underbrace{\frac{\alpha_{i}\cdot \mu \cdot \beta }{\left(p\cdot d_{i}\;+\frac{1}{D_{n}}\right)}}},\;R_{0}^{2}=\underset{\mathrm{pre}-\mathrm{isolated}}{\underbrace{\frac{\left(1-\alpha_{i}\right)\cdot \beta \cdot (1-c)}{\left(p\cdot d_{i}\;+\frac{1}{D_{p}}\right)}}}+\underset{\mathrm{non}-\mathrm{isolated}}{\underbrace{\frac{\alpha_{i}\cdot \mu \cdot \beta \cdot (1-c)}{\left(p\cdot d_{i}\;+\frac{1}{D_{n}}\right)}}}$ (see details in Supplementary data). The numerators are the expected numbers of secondary infections per day, and the denominators are the sums of removal rates from each infected compartment, where $p\cdot d_{i}$ is the daily detection rate.

Let $S^{*},{E^{*},\;I}^{n*},\;I^{p*},\;I^{s*},\;R^{*}\;$be the numbers of hosts in the $S,{E,\;I}^{n},\;I^{p},\;I^{s},\;R$ compartments at the disease-free equilibrium. The disease-free equilibrium ($I^{n*}=I^{p*}=I^{s*}=0$) is locally unstable [30] to the introduction of new exposed or infected individuals if $R_{0}^{i}>1$ for i=1 or i=2. Using parameters adjusted to realistic SARS-CoV-2 values (Table 1), and specifically a transmission rate β≥0.35, we ensure that the disease-free equilibrium in our model is locally unstable, allowing the outbreak of the epidemic for both strains.

**Table 1.** Model parameters with estimated values

Parameter	Description	Estimate	Source
N	Total population size	8 804 190	NYC population size (2020), US Census Bureau [31]
β	Transmission rate in pre-isolated infected individuals	0.35–1.2	Li *et al*. [2]
$\alpha_{1},\alpha_{2}$	Fraction of non-isolated infections	0.35	Sah *et al*. [14]
μ	Relative infectiousness of non-isolated infected individuals	0.65–0.75	Byambasuren *et al*. [15]
$D_{p}$	Number of days in the pre-isolated phase	3	Casey *et al*. [32]
$D_{n}$	Number of days in the non-isolated phase	6	Byrne *et al*. [33]
$D_{s}$	Number of days in the isolated phase	14	Byrne *et al*. [33]
Z	Length of incubation period, or number of days in the exposed phase	5	McAloon *et al*. [34]
p	Daily tests per thousand people	0.01–25	Coronavirus (COVID-19) Testing—Statistics and Research—Our World in Data [35]. Accessed 30 December 2020
$d_{1},d_{2}$	Detectability of detectable and test-evasive strain, respectively (true positive rate)	*d*_1_ = 0.9	Arevalo-Rodriguez *et al*. [20, 21]

**Figure 2.**
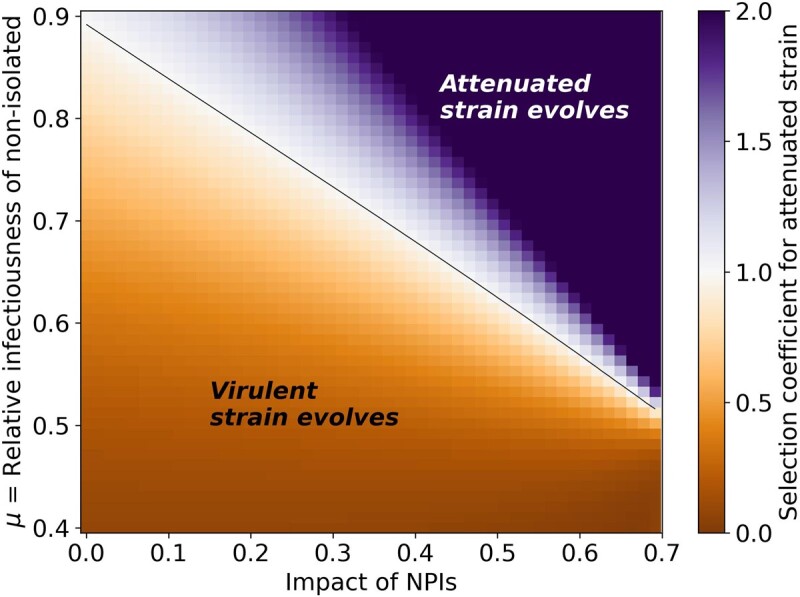
Effective NPIs facilitate the evolution of the attenuated virus. This figure presents the conditions for evolution of either the attenuated or the virulent strains under constant NPIs. High impact NPIs (right side) facilitate the evolution of the attenuated strain, while low impact NPIs (left side) facilitate the evolution of the virulent strain. The attenuated strain can also evolve if non-isolated individuals are infectious enough relative to pre-isolated individuals (μ is high, top side). Here, $\alpha_{1}=0.35$, $\alpha_{2}=0.95$.

**Figure 3.**
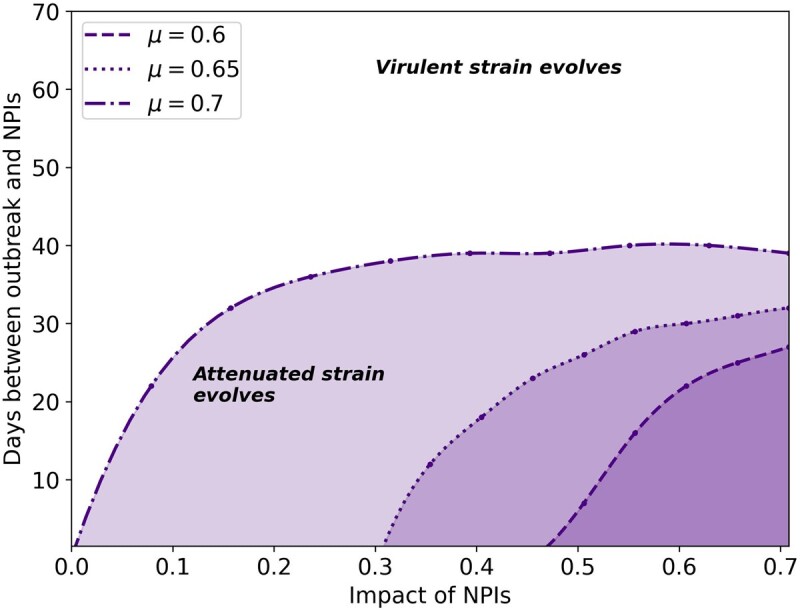
Earlier start of NPIs favors the evolution of the attenuated strain. Each curve corresponds to a different relative transmission rate by non-isolated individuals, μ. The colored areas below each curve show the regions in which the attenuated strain evolves. The areas above each of the curves show the regions in which the virulent strain evolves. Here, $\alpha_{1}=0.35$, $\alpha_{2}=0.95$.

$R_{0}^{i}\;$ can be used to quantify the expected number of secondary cases when a viral strain infects a single host in an otherwise fully susceptible population. In the basic model, $R_{0}^{2}>R_{0}^{1}$ if $\mu \cdot D_{n}>D_{p}$. Substituting with the model parameters (Table 1), this is equivalent to μ>0.5. This is a necessary but insufficient condition for the attenuated strain to evolve [36] (Fig. 1). Since we examine a system where the two strains ‘compete’ over the same finite population of susceptible individuals, cannot be determined solely from the reproductive number [37, 38]. In this case, the difference in the early spread of each strain at the onset of the epidemic [38] can determine the winning strain (Figs 1–3).

### Numerical solution

To analyze the model, we use parameter values estimated from COVID-19 literature (Table 1) and solve Equations (1)–(6) numerically using Python with NumPy and SciPy [39, 40]. For the initial conditions, we assume a population of mostly susceptible individuals $\left(S_{0}∼N\right)$, with a small number of exposed individuals, divided equally between the two strains (the attenuated strain can also evolve from rarity, see Supplementary Fig. S1). New hosts are not introduced, so after enough time has passed, the population reaches a disease-free equilibrium ($I^{n*}=I^{p*}=I^{s*}=0$). At this equilibrium, $S^{*}+R_{1}^{*}+R_{2}^{*}=N$, meaning all individuals are either susceptible or have recovered from the disease.

### Competition coefficient

Note that the total number of hosts infected by strain i during the entire duration of the epidemic is the number of hosts recovered from strain i at the disease-free equilibrium, $R_{i}^{*}$. Thus, we compute the ratio of $R_{i}^{*}\;$and the initial number of hosts infected with strain i, $W_{i}=\frac{R_{i}^{*}}{I_{i}^{n}(0)+I_{i}^{p}(0)+I_{i}^{s}(0)+E_{i}(0)}$*,* which is equivalent to the Wrightian fitness [41] of strain i. The competition coefficient of the attenuated strain is defined to be $w=W_{2}/W_{1}$. This competition coefficient is equivalent to the relative fitness of the attenuated strain compared with the virulent strain. When w>1, the attenuated strain outcompetes the virulent strain, and so the frequency of the attenuated strain increases; when w<1, the virulent strain outcompetes the attenuated strain. That is, the attenuated strain is expected to evolve when w>1.

## RESULTS

### Attenuation

We first consider competition between a virulent strain and an attenuated strain, where the attenuated strain has a lower fraction of symptomatic cases compared with the virulent strain ($\alpha_{2}<\alpha_{1}$). The two strains ‘compete’ for the same population of susceptible hosts. We define ‘effective transmission rate’ for each of the strains such that $\beta_{\mathrm{eff}}^{i}=\alpha_{i}\cdot \mu \cdot \beta +(1-\alpha_{i})\cdot \beta$. We model the impact of NPIs as a reduction in the transmission rate, and a higher impact of NPIs is associated with a lower transmission rate (see Supplementary data). The difference in effective transmission rates between the two strains is $\Delta \beta_{\mathrm{eff}}=\beta_{\mathrm{eff}}^{1}-\beta_{\mathrm{eff}}^{2}=\beta \cdot \left(\alpha_{2}-\alpha_{1}\right)\cdot \left(1-\mu \right).$ Under the assumptions of this analysis (${\alpha_{1}<\alpha }_{2},\;\mu <1$), $\Delta \beta_{\mathrm{eff}}\;$is always positive. Because the attenuated strain benefits from a higher fraction of non-isolated hosts, $\alpha_{2}$, it has more opportunities for transmission compared with the virulent strain, as infected hosts are less likely to be isolated. The disadvantage of the attenuated strain is a lower effective transmission rate, as the relative transmission rate of non-isolated hosts is lower compared with pre-isolated hosts (μ<1). We consider constant NPIs as a fixed reduction in the transmission rate (β) during the entire intervention. Thus, when the impact of NPIs on the transmission rate is weak, the virus spreads rapidly, and the susceptible population is quickly infected by the more virulent Strain 2. In contrast, when the impact of NPIs on the transmission rate is strong, the epidemic lasts longer (i.e. the curve is ‘flattened’), allowing the attenuated Strain 1 more time to spread. Additionally, when the transmission rate is reduced, the difference in the effective transmission rates between the two strains $\Delta \beta_{\mathrm{eff}}$ is smaller. Indeed, Fig. 2 shows that selection for the attenuated strain increases with the impact of NPIs and with the relative transmission rate of the attenuated strain (μ). We note that for a given μ and impact of NPI, the threshold for evolution of each strain (Fig. 2, contour line) is independent of $\alpha_{2}$, the fraction of non-isolated infections caused by the attenuated strain (Supplementary Fig. S2).

NPIs have been implemented with various schedules, determined by epidemiological metrics [42], economic pressures [42], public opinion [42] and in some cases have probably started later than planned [43]. Therefore, we explored the effects of temporal application of NPIs on the competition between attenuated and virulent strains. In our analysis, the reproductive number ($R_{0}$) for the attenuated strain is higher than for the virulent strain, but the effective transmission rate is lower. Since the two strains are competing over the same finite population of susceptible individuals, the competition can be determined by an advantage in the number of infected during the early stages of the epidemic. Figure 3 shows the effect of the number of days between the outbreak and start of NPIs on the competition between the two viral strains. Overall, if NPIs are implemented earlier, then the attenuated strain is more likely to evolve. As above (Fig. 2), weaker NPIs favor the virulent strain, while stronger NPIs favor the attenuated strain.

**Figure 4.**
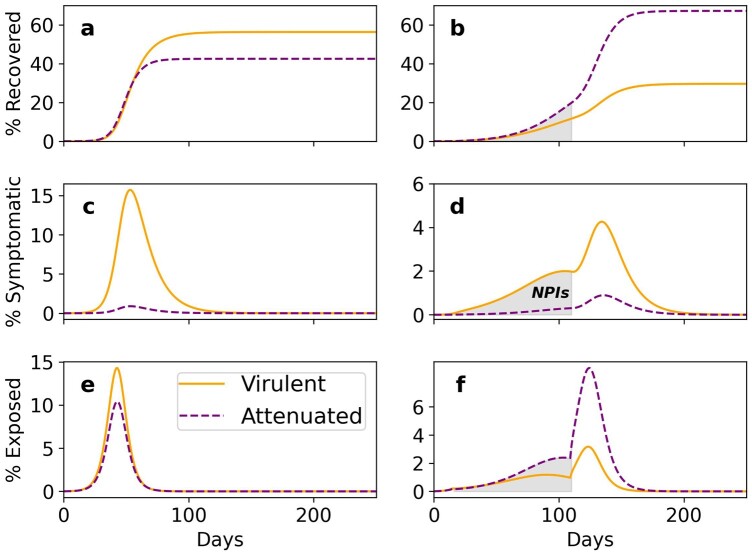
Temporal application of NPIs favors the evolution of the attenuated strain. Without NPIs (left), the virulent strain takes over the viral population. When NPIs are applied (right, shaded area), the virulent strain is more frequent among isolated (**d**), but the attenuated strain becomes more frequent in exposed individuals during the NPIs (**f**), and after the NPIs end it is the dominant strain (**b**). Parameters: NPIs start on day 31 and end on day 150. μ=0.6. Impact of NPIs = 0.65.

Figure 4 compares the dynamics without NPIs (left) and with NPIs that begin a certain number of days after the outbreak and are lifted after a limited time (right). We find that NPIs significantly affect the competition between the two strains, changing the direction of selection on the virus and leading to the evolution of the attenuated strain (compare Fig. 4a and b). While NPIs reduce the peak number of isolated cases (compare Fig. 4c and d), a ‘second wave’ of infections may occur when NPIs are over (Fig. 4d and f). Here, this ‘second wave’ is dominated by the attenuated strain (Fig. 4f) that produces more non-isolated infections compared with the virulent strain (compare purple dashed line and orange solid line in Fig. 4d).

### Test evasion

We consider the evolution of a test-evasive strain. *Detectability* is defined here as the test sensitivity, or the true positive rate: the probability that an infected individual will be correctly detected by a single test. The detectability of the detectable and test-evasive strains is $d_{1},\;d_{2}$ respectively, where we assume $d_{2}<d_{1}$. All else being equal, the test-evasive strain will evolve due to its lower detectability (Supplementary Fig. S3a). However, lower detectability likely incurs a cost for the virus, as the target sequences for SARS-CoV-2 tests are in regions essential for replication and other critical aspects of the viral life cycle [22]. We assume this cost, c, is expressed in a decreased transmission rate, such that $\beta_{2}=(1-c)\cdot \beta_{1}$ (see Supplementary Equation S2.1). Thus, we examine a competition between a detectable and a test-evasive strain that is less transmissible compared with the virulent strain, c>0.

**Figure 5.**
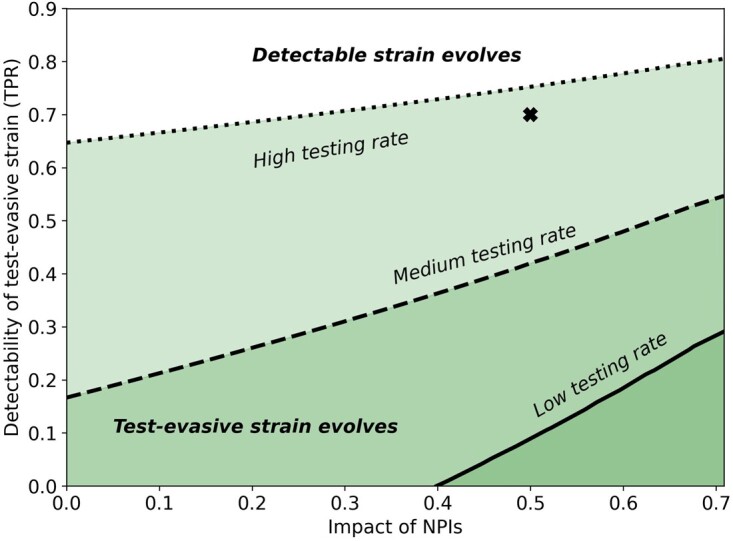
High testing rate and effective NPIs favor the evolution of test-evasive strains. Conditions for the evolution of test-evasive strains despite a cost of transmission (see Supplementary Fig. S3b for further decreased transmission). Each line corresponds to a different testing rate, p (p=4, 6.3, 15 per 1K people for low, medium and high testing rate, respectively). The colored areas below each line show the regions in which the test-evasive strain evolves. The areas above each of the lines show the regions in which the detectable strain evolves. Test-evasive strains can also evolve if the detectability of the test-evasive strain is low enough: given that the impact of NPIs is 0.5, a test-evasive strain with detectability $d_{2}=0.7$ (marked with ‘X’) would evolve when the testing rate is high, but the more detectable strain would evolve when testing rate is low or medium. Here, $\alpha_{1}=0.65,\;\alpha_{2}=0.65,\;\mu =0.6,\;c=0.01,d_{1}=0.9$.

Figure 5 shows that a higher testing rate (*p*) may select for a test-evasive strain, even when reduced detectability incurs decreased transmission. When the impact of NPIs is stronger (right), the test-evasive strain evolves even when the testing rate is low (solid line).

**Figure 6.**
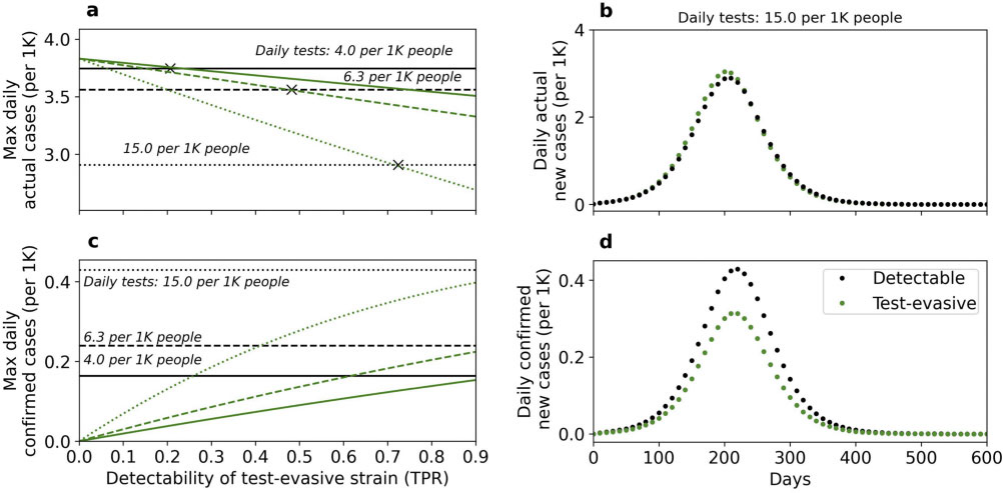
Effects of decreased detectability on epidemiological metrics. These results demonstrate the outcomes of two separate outbreaks: (i) exclusively by a detectable strain (darker lines) and (ii) exclusively by a test-evasive strain (lighter lines), under three testing regimes (solid, dashed and dotted lines). (**a**, **c**) The maximum number of actual and test-confirmed daily cases, respectively. (**b**, **d**) A timeline of these epidemiological metrics for a relatively high testing rate and low detectability. Higher detectability of the test-evasive strain increases the daily number of confirmed cases (c, lighter lines) and decreases the daily number of actual cases (a, lighter lines). The effect of detectability on the number of daily actual cases is associated with the cost of infectiousness, c, producing detectability thresholds (a, marked with ‘X’), above which the number of actual daily cases for the test-evasive strain is lower than for the detectable strain (see Supplementary Fig. S4 for the effect of decreased detectability without cost of transmission). A higher testing rate increases the number of confirmed cases (c, compare solid, dashed and dotted lines) and decreases the number of actual cases (a, compare solid, dashed and dotted lines). Overall, when testing rate is high and the epidemic is driven by a test-evasive strain with low detectability (b, d), the number of confirmed cases is lower compared with an epidemic driven by a detectable strain, while the number of actual cases is higher. Here, Impact of NPIs = 0.65, $\mu =0.6,\;\alpha_{1}=0.65,\alpha_{2}=0.65,d_{1}=0.9,\;c=0.01.$ For (b) and (d), $d_{2}=0.6,\;p=15$ per 1K people.

In Fig. 6, we examine the effects of decreased detectability on epidemiological metrics. We explore two scenarios of epidemic outbreaks: exclusively by a detectable strain (black lines), and exclusively by a test-evasive strain (green lines). The number of confirmed daily cases increases with the testing rate (Fig. 6c, compare solid, dashed and dotted lines), while the number of actual daily cases decreases (Fig. 6a, compare solid, dashed and dotted lines). When the epidemic is driven by a test-evasive strain (green lines), the daily number of confirmed cases and daily percent of positive tests (Supplementary Fig. S5) increase with the detectability of the test-evasive strain (Fig. 6c), while the daily number of actual cases decreases (Fig. 6a). Overall, when testing rate is high and the epidemic is driven by a test-evasive strain with low detectability (Fig. 6b and d), the number of confirmed cases can be misleading: it is significantly lower for the test-evasive strain, despite the number of actual cases being higher.

## DISCUSSION

We examined the expected selection pressures exerted by NPIs and testing on disease severity and detectability of SARS-CoV-2. We found that when stronger NPIs are applied, selection may act to reduce disease severity. Additionally, the timely application of NPIs could significantly affect the competition between viral strains, favoring the milder strain. Furthermore, a higher testing rate can select for a test-evasive viral strain, even if that strain is less transmissible than the competing, more detectable strain. Our results also show that if a test-evasive strain evolves, reductions in epidemiological metrics such as confirmed daily cases may be due to reductions in test sensitivity rather than reductions in the actual number of cases.

Our model makes several simplifying assumptions. We assume that individuals exhibiting clinical symptoms are isolated and therefore do not transmit the virus. However, such individuals may still be able to infect others, whether it is in a medical facility, within the household, or due to non-compliance with isolation guidelines. Nevertheless, practical strategies have been put in place to reduce nosocomial transmission [44], and evidence suggests that the overall risk of hospital-acquired COVID-19 is low [44]. In our model, when the impact of NPIs is low, the susceptible population is infected rapidly by the more virulent strain. Our model could be extended by allowing individuals that recovered from one strain to become infected with another strain, and in that case the attenuated strain may evolve even when the impact of NPIs is weak. We assume that the entire population is tested daily with a testing rate estimated by realistic parameters (Figs 5 and 6). However, the average testing rate likely underestimates the rate for infected cohorts, as individuals who experience symptoms or have been exposed to a confirmed case are more inclined to be tested. Applying a higher testing rate would make the evolution of the test-evasive strain even more likely (Fig. 5).

The emergence of novel SARS-CoV-2 variants has raised widespread concern [9]. SARS-CoV-2 is likely a pathogen of recent zoonotic origin, and can therefore further adapt to its new human host [45]. While adapting to its new host, the virus may explore new evolutionary paths, possibly including paths where the virus can become more virulent without significant costs. Previous models of the evolution of SARS-CoV-2 have suggested that the emergence of new variants should be expected [9], and that interventions, such as social distancing [45] and vaccination [9] could shape the evolutionary trajectory of the virus. Models predict that as long as circulating strains cannot infect recovered or immunized individuals, selection could shift to favor prolonged infectious periods rather than increased transmissibility [9], and could also affect virulence [9, 45]. Some existing mutations have been said to increase infectiousness [46]. It has been suggested that the Omicron variant causes a milder disease [47], while it is also suspected of ‘immune escape’, eluding the human immune response [48], such that more recovered individuals remain susceptible to reinfection and possibly causing a reduction in the effectiveness of vaccines [48]. The future evolutionary and epidemiological trajectories of the virus are difficult to predict [49, 50], and it may evolve into variants differing in their disease severity and transmissibility. Our results show that NPIs and testing policies, primarily designed and applied to control the spread of the pandemic, may also steer the evolution of the virus towards attenuation and test-evasion.

## SUPPLEMENTARY DATA

Supplementary data is available at *EMPH* online.

## Supplementary Material

eoac013_Supplementary_Data
